# CFTR Deficiency Affects Glucose Homeostasis via Regulating GLUT4 Plasma Membrane Transportation

**DOI:** 10.3389/fcell.2021.630654

**Published:** 2021-02-15

**Authors:** Junzhong Gu, Weiwei Zhang, Lida Wu, Yuchun Gu

**Affiliations:** ^1^Molecular Pharmacology Laboratory, Institute of Molecular Medicine, Peking University, Beijing, China; ^2^Peking-Tsinghua Center for Life Sciences, Peking University, Beijing, China; ^3^Translational and Regenerative Medicine Centre, Aston Medical School, Aston University, Birmingham, United Kingdom

**Keywords:** cystic fibrosis, CFTR, CF-related diabetes, glucose homeostasis disorder, GLUT4

## Abstract

Cystic Fibrosis (CF) is an autosomal recessive disorder caused by mutations in the Cystic Fibrosis Transmembrane Conductance Regulator (CFTR) gene. CF-related diabetes (CFRD) is one of the most prevalent comorbidities of CF. Altered glucose homeostasis has been reported in CF patients. The mechanism has not been fully elucidated. Besides the consequence of pancreatic endocrine dysfunction, we focus on insulin-responsive tissues and glucose transportation to explain glucose homeostasis alteration in CFRD. Herein, we found that CFTR knockout mice exhibited insulin resistance and glucose tolerance. Furthermore, we demonstrated insulin-induced glucose transporter 4 (GLUT4) translocation to the cell membrane was abnormal in the CFTR knockout mice muscle fibers, suggesting that defective intracellular GLUT4 transportation may be the cause of impaired insulin responses and glucose homeostasis. We further demonstrated that PI(4,5)P_2_ could rescue CFTR related defective intracellular GLUT4 transportation, and CFTR could regulate PI(4,5)P_2_ cellular level through PIP5KA, suggesting PI(4,5)P_2_ is a down-stream signal of CFTR. Our results revealed a new signal mechanism of CFTR in GLUT4 translocation regulation, which helps explain glucose homeostasis alteration in CF patients.

## Introduction

Cystic fibrosis (CF) is one of the most common autosomal recessive disorders in the Caucasian population. It is caused by mutations in the CFTR gene. CF-related diabetes (CFRD) affects 19% of adolescents with CF (Moran et al., 2010), 50% adults CF patients (Moran et al., 2009, 2010), indicating that CFRD is a progressive complication of CF. Despite advances in therapies designed to address the disease’s symptoms, the death rate is still high. As a result, it is crucial to study the mechanism of CFRD thoroughly.

One of the prevailing mechanistic beliefs is that CFRD results from defective insulin secretion of pancreatic β-cell, which is due to the combination of chronic pancreatitis and eventual loss of the islet cells (Cucinotta et al., 1994; Marshall et al., 2005; Costa et al., 2007; Mohan et al., 2009; Guo et al., 2014). Autopsy data confirmed the eventual loss of islet tissue and decreased β-cell numbers in aged CF patients (Iannucci et al., 1984; Lohr et al., 1989). However, most CF patients demonstrate abnormalities in glycaemic control regardless of the class and severity of the CFTR mutation (Wooldridge et al., 2015). Of the thousand CFTR mutations that have been identified, approximately 20 are understood to be disease-causing and are categorized into five classes of mutations of increasing disease severity (Manderson Koivula et al., 2017). CFTR in the regulation of insulin secretion and β cell function has also been reported as the mechanism of CFRD by several recent studies (Stalvey et al., 2006; Edlund et al., 2014; Guo et al., 2014; Fontes et al., 2015), which cannot fully explain the impaired insulin responses and glucose homeostasis reported in CF patients. In addition to the traditional view that CFRD is the consequence of pancreatic endocrine dysfunction, we speculated that CFRD might also affect insulin-responsive tissues’ function.

Several studies found that CF patients with overt diabetes exhibited peripheral and hepatic insulin resistance (Moran et al., 1994; Holl et al., 1995; Hardin et al., 2001; Fontes et al., 2015; Boudreau et al., 2016). However, little is known regarding the mechanism of CFTR loss-of-function in insulin resistance. Insulin facilitates the entry of glucose into muscle, adipose, and several other tissues. The typical mechanism by which cells can take up glucose is by facilitated diffusion through a family of hexose transporters. Skeletal muscle provides the most substantial contribution (∼70%) of insulin-dependent glucose disposal, and this process is mediated by the insulin-responsive glucose transporter 4(GLUT4) (Bouche et al., 2004; Leto and Saltiel, 2012). As a result, we speculated that CFTR deficiency might result in impaired insulin action and GLUT4 dysfunction in skeletal muscle. In this study, we found that CFTR knockout mice exhibited altered glucose homeostasis, and the reason was defective intracellular GLUT4 transportation.

## Materials and Methods

### Cell Culture and Transfection

Flexor Digitorum Brevis (FDB) muscle fibers isolation from mice skeletal muscle was previously described (Park et al., 2014). Mouse skeletal muscle cell lines, C2C12 myoblasts, were cultured in DMEM (GIBCO #11965) supplemented with 10% FBS (HyClone #30071.03). C2C12 myotubes differentiation was then induced by switching the medium to DMEM supplemented with 2% HS (GIBCO #26050070). The medium was changed every 24 h. The plasmid of myc–GLUT4–EGFP, bearing the c-Myc epitope tag in the first extracellular loop and GFP at the carboxyl terminus, was purchased from addgene (#52872). According to the manufacturer’s instructions, the plasmid was transfected into HEPG2 and Hela cells using Lipofectamine 2000 (Invitrogen). C2C12 myoblasts and myotubes were transfected with adenovirus (Vigenebio, China) to express myc–GLUT4–EGFP protein.

### Immunofluorescent Analysis

Cells that express myc–GLUT4–EGFP were cultured with media supplemented with 1% FBS overnight and then were treated with insulin (100 nM) for 30 min. Cells were then fixed with 4% paraformaldehyde, followed by immunocytochemistry using the anti-Myc antibody (Beijing TDY Biotech LTD, #TDY002) and Cy3 -conjugated anti-mouse IgG antibody (Beijing CWBIO, # CW0145S). The cells were imaged with a spin disk confocal system (Revolution XD; ColdSpring Science Corporation) with a CSU-X1 confocal head (Yokogawa) mounted on an inverted microscope (IX81ZDC2; Nikon) with Perfect Focus, using an EMCCD camera (iXon3 DU-897D-C00-#BV; Andor). MetaMorph controlled image acquisition and all other peripherals.

### Animal Experiments

All methods were carried out following relevant guidelines and regulations. All experimental protocols were approved by the University Ethics Committee, Institute of Molecular Medicine, Peking University. All animals received humane care in compliance Guide for the Institutional Animal Care and Use Committee (IACUC). CFTR knockout (KO) and heterozygote (HET) mice were purchased from Jackson Lab(CFTR S489X-) and bred to generate homozygous mutants (KO), heterozygote (HET), and wild-type (WT) mice. CFTR KO mice were genotyped by standard PCR according to the genotyping protocol. Mice were housed under controlled temperature (21°C) on a 12 h light-dark cycle with unrestricted access to food and water.

FABP-hCFTR-CFTR transgenic mice harbor the FABP-hCFTR transgene and a targeted knock-out mutation of the CFTR. This model may be used to assess the effects of the null mutation and may be useful as a mouse model of severe phenotype cystic fibrosis.

### Glucose Tolerance Test, Insulin Tolerance Tests, and Determination of Insulin Levels *in vivo*

For glucose tolerance tests (GTTs), the mice were starved overnight and were given 2.5 g/kg body weight of glucose by IP injection. Blood was then taken from the tail vein before and 15, 30, 60, and 120 min after injection to determine blood glucose by using an Accu-Chek blood glucose monitor (Roche). To determine insulin plasma levels, blood was collected, and the plasma was obtained, and analysis was performed with an insulin ELISA (Millipore). For ITT, 5 h fasted mice were given human insulin (0.75 U/kg, Novolin) by IP injection. The blood glucose concentration was monitored before and after 15, 30, 60, and 120 min after injection. During GTT and ITT, mice were caged with blinded identity and random orders.

### Immunoblotting

The expression and phosphorylation of each protein were analyzed by Western blot analysis. The membrane protein was extracted by using a membrane protein extraction kit (Merck Millipore). The protein was quantified using the Bradford reagent (BioRad). Before loading into the 10% SDS-PAGE, the samples were mixed with a 5x loading buffer and boiled at 98°C for 5 min. After gel electrophoresis, the proteins were then transferred to a PVDF membrane (0.45 μm, Merck Millipore) under 250 mA for 90 min. The membranes were incubated with a blocking buffer (5% non-fat milk in Tris-buffered saline containing 0.5% Tween-20) for 1 h at room temperature and then incubated with the indicated primary antibodies at 4°C overnight. The membrane was incubated with horseradish peroxidase-conjugated secondary antibody for 1 h at room temperature. The target protein bands were visualized using Amersham Imager 600 (GE healthcare).

Rabbit polyclonal to GLUT4(ab654,1:2,000), Rabbit polyclonal to PIP5K1A(ab654,1:1,000), and Mouse monoclonal to alpha 1 Sodium Potassium ATPase(ab7671, 5 μg/ml) were purchased from Abcam (United Kingdom); Mouse Monoclonal Antibody to β-tubulin(TDY041, 1:1,000), Mouse Monoclonal Antibody to β-actin(TDY045, 1:1,000), Goat Polyclonal Antibody to Mouse IgG (H + L), HRP Conjugated(S001, 1:1,000), and Goat Polyclonal Antibody to Rabbit IgG (H + L), HRP Conjugated(S004, 1:1,000) were from TDY(Beijing, China).

### Dot-Blot Analysis

PI(4,5)P_2_ were measured by dot-blot analysis. C2C12 myotubes were treated with CFTR (inh)-172(Chemegen) for 24 h. FDB muscle fibers isolated from CFTR KO, HET, WT mice. The whole-cell lysates were then extracted from C2C12 myotubes, and FDB muscle fibers were blotted onto nitrocellulose membranes (Bio-Rad Laboratories). These were probed with PI(4,5)P_2_ antibody (Abcam, # ab11039) at a 1:500 dilution and detected using a horseradish peroxidase-conjugated secondary antibody (Beijing TDY Biotech LTD, #E009) and were visualized using Amersham Imager 600 (GE healthcare). Experiments were repeated at least three times.

### [^3^H]2-Deoxy-D-Glucose Uptake Assay

Uptake of [^3^H]2-deoxyglucose (Sigma, 73698-45-0) was measured in C2C12 myocytes differentiated in 24-well plates. After differentiation, cells were washed twice with DMEM (GIBCO #11965) and incubated in the DMEM for 2 h at 37°C. Cells were then washed twice in PBS and then incubated in KRBB (G-clone, RS1800) in the presence or absence of insulin for 30 min at 37°C. CFTR (inh)-172 was added 30 min before insulin. After insulin treatment, uptake of 10 μM [^3^H]2-deoxyglucose was measured for 15 min at 37°C. Reactions were terminated by rapidly washing the cells twice with cold KRBB. Cells were then were lysed in 0.1 N NaOH with SDS 0.1%, and radioactivity was determined by liquid scintillation counting and normalized according to the total protein content. Non-specific uptake was determined in the presence of 20 μM cytochalasin B.

The mice’s skeletal muscle was quickly dissected after killing the mice. Before the experiment, mice have fasted for 12 h. Do not disturb the mice and keep them in a resting state. The muscles were incubated in KRBB in the presence or absence of insulin for 30 min at 37°C, uptake of 10 μM [^3^H]2-deoxyglucose was measured for 15 min at 37°C, as described above.

### Statistical Analysis

All statistical calculations were carried out using Graphpad Prism 6. Data are expressed as average ± SEM of at least three independent experiments. The statistical significance was determined using a *t*-test when comparing two groups and ANOVA when comparing multiple groups. A value of *P* < 0.05 was considered statistically significant.

## Results

### CFTR Dysfunction Affects Mice’s Glucose Homeostasis and Body Weight

**FIGURE 1:**
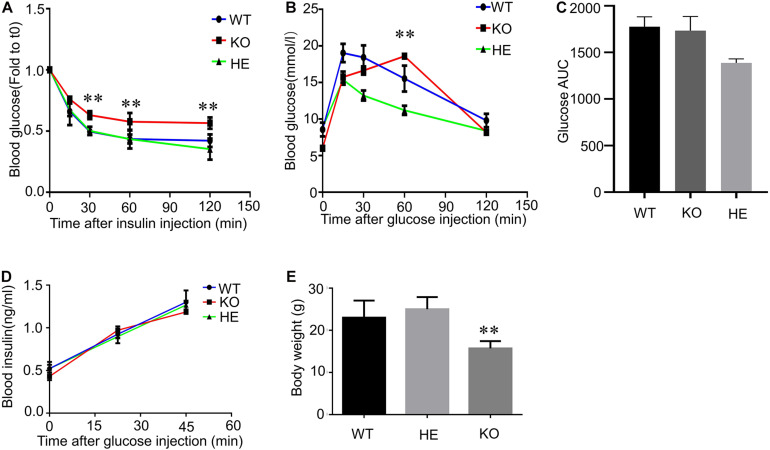
Insulin resistance and Glucose tolerance in CFTR knockout mice. **(A)** Intraperitoneal insulin tolerance tests were performed in 20-weeks-old male mice WT, HET, and CFTR KO mice. **P < 0.01; NS, not significant. **(B)** OGTTs were performed in 20-weeks-old male mice WT, HET, and CFTR KO mice. **P < 0.01; NS, not significant. **(C)** Area under the curve (AUC) of changes in blood glucose levels. **(D)** Following an injection of 250 mg/ml glucose, serum insulin concentration was measured by ELISA. NS, not significant. **(E)** Bodyweight was measured in 20-weeks-old male mice WT, HET, and KO mice. Data are mean ± SEM of 7–9 mice per group. **P < 0.01 vs. WT.

To investigate the connection between CFTR and glucose homeostasis, we performed insulin tolerance tests and glucose tolerance tests in 20-weeks-old male WT, heterozygous(HET), and CFTR KO mice (Figures 1A,B). During intraperitoneal insulin tolerance tests, the decrease in blood glucose was less marked in CFTR^–/–^ mice than that of WT and HET mice following 10 mg/ml insulin injection (Figure 1A), suggesting insulin resistance. Moreover, the blood glucose level in CFTR^–/–^ mice was also much higher than that of WT and HET mice 2 h after insulin injection (Figure 1A). It took CFTR^–/–^ mice 60 min to reach the blood glucose summit in response to glucose tolerance tests, while it only took 15 min in WT and HET mice(Figure 1B). However, the glucose AUC was similar among WT and KO groups but slightly lower in the HE group (Figure 1C), indicating that the delay in attaining peak glucose levels during the OGTT suggests delayed glucose absorption. Reduced first, second, and amplifying phase secretion in CF islets have been reported by numerous researchers (Sun et al., 2017; Kelly et al., 2019); however, it was found that serum insulin level of CFTR^–/–^ mice showed no significant difference than WT and HET mice by ELISA (Figure 1D), indicating that the amount of insulin secretion by CFTR^–/–^ mice was normal before CFRD. Notably, maintained in a stable environment with standard chow and water, CFTR^–/–^ mice’s average body weight was much lower than WT and HET mice (Figure 1E). We suspected that the lower body weight of CFTR^–/–^ mice resulted from inadequate glucose intake, but other possibilities include CFTR-related glucosuria or increased metabolic rate, requiring further confirmation. In conclusion, CFTR dysfunction affects mice’s glucose homeostasis and body weight.

### CFTR Is Essential to Insulin-Induced GLUT4 Cell Membrane Translocation and Glucose Uptake

**FIGURE 2:**
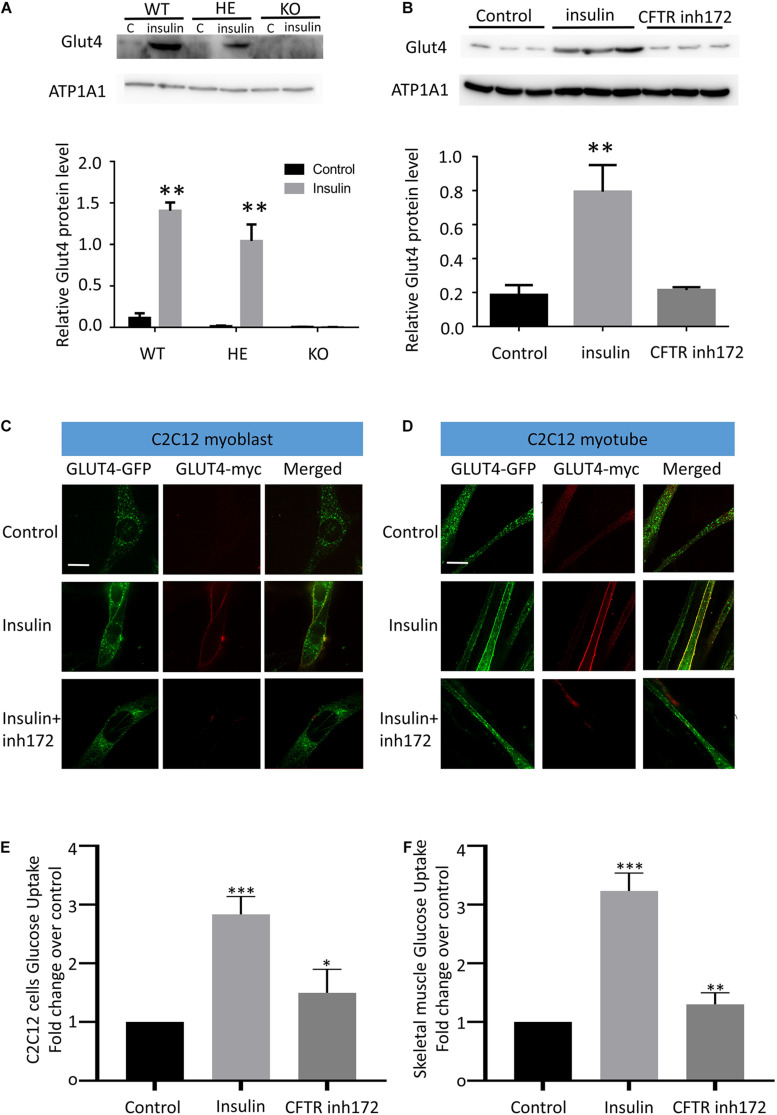
CFTR regulates GLUT4 membrane translocation and glucose homeostasis. **(A)** The membrane localization of GLUT4 in CFTR KO mice FDB muscle after injection of 10 mg/ml insulin was measured by Western blot. Images are representative of triplicate experiments with similar results. **P < 0.01 compared with control (*n* = 3). **(B)** The membrane expression of GLUT4 in C2C12 myotubes was measured by Western blot. **P < 0.01 compared with control (*n* = 3). **(C)** myc-GLUT4- GFP was transfected into C2C12 myoblasts by adenoviruses. The cells were stimulated with insulin (100 nM) for 30 min. GLUT4 translocation to the plasma membrane was detected by confocal microscopy. Glut4-GFP(green), Glut4-myc(red). Scale bar, 10 μm. **(D)** GLUT4 translocation to the plasma membrane of differentiated C2C12 myotubes was detected by confocal microscopy. Glut4-GFP(green), Glut4-myc(red). Scale bar, 10 μm. **(E)** The glucose uptake assay of C2C12 cells. Data are mean ± SD values for two independent assays performed in triplicate. ***P < 0.001, *P < 0.05. **(F)** The glucose uptake assay of Flexor Digitorum Brevis muscle fibers cells. Data are mean ± SD values for two independent assays performed in triplicate. ***P < 0.001, **P < 0.01.

When GLUT4 glucose transporters are present in cytoplasmic vesicles, they are useless for transporting glucose. The binding of insulin to receptors on such cells leads rapidly to the fusion of those vesicles with the plasma membrane and the insertion of the glucose transporters, thereby giving the cell the ability to take up glucose efficiently. As a result, glucose uptake depends on insulin-stimulated membrane translocation of the GLUT4. To investigate the relation between CFTR and GLUT4, GLUT4 cell membrane expression was measured by western blot in mice skeletal muscle fibers and C2C12 myotubes. The Flexor Digitorum Brevis (FDB) muscle fibers total membrane protein were immediately isolated from WT, HET, and CFTR KO mice 30 min after insulin injection stimulation. Evident membrane translocation of GLUT4 was found in WT and HET mice muscle fibers with insulin stimulation (Figure 2A). However, there was no membrane expression of GLUT4 in CFTR^–/–^ mice with or without insulin stimulation (Figure 2A). Consistent with this, the insulin-stimulated GLUT4 cell surface translocation was also blocked by a specific CFTR inhibitor [CFTR (inh)-172] in differentiated C2C12 myotubes (Figure 2B). To directly show the translocation of GLUT4, C2C12 myoblasts and myotubes were transiently transfected with myc–GLUT4–EGFP fusion plasmids. Myc epitope was inserted in the first exofacial loop of GLUT4 N terminus and presented outside of the cell membrane, which could be detected by the antibody without perforation. C2C12 Cells were then fixed (without perforation) and labeled with an anti-Myc antibody followed by Texas Red secondary to track the membrane translocation of GLUT4 (red). We found that GLUT4 was expressed in the cytoplasm (Figures 2C,D, Control), and insulin could induce translocation of GLUT4 to cell membranes both in C2C12 myoblasts and myotubes (Figures 2C,D). However, this process could be blocked by CFTR (inh)-172 (Figures 2C,D). The effects of CFTR (inh)-172 on insulin-stimulated glucose uptake activity were also tested in differentiated C2C12 myotubes and freshly isolated Flexor Digitorum Brevis (FDB) muscle fibers cells. We found that CFTR (inh)-172 significantly decreased 2-DG uptake in insulin-stimulated C2C12 myotubes (Figure 2E) and freshly isolated Flexor Digitorum Brevis (FDB) muscle fibers cells (Figure 2F).

Some tissues, such as the liver, do not require insulin for efficient uptake of glucose; this is because these cells do not use GLUT4 to import glucose, but rather, GLUT1 and GLUT2 are not insulin-dependent. We wondered if similar results could be observed in liver cells. HEPG2 cells were transiently transfected with myc–GLUT4–EGFP fusion plasmids. HEPG2 Cells were then fixed (without perforation) and labeled with an anti-Myc antibody followed by Texas Red secondary to track the membrane translocation of GLUT4 (red). It was found that CFTR (inh)-172 could also block insulin-induced GLUT4 membrane translocation in HEPG2 (Supplementary Figure S1A) and Hela cells (Supplementary Figure S1B). To further confirm CFTR’s role on GLUT4 translocation, as Hela cells endogenously express CFTR (Billet et al., 2017), we knocked out CFTR in Hela cells by CRISPR/Case9 (Supplementary Figure S1C). It was found that insulin could no longer induce GLUT4 translocation to the cell membrane in CFTR^–/–^ cells (Supplementary Figure S1D). Taken together, these results suggested that CFTR was required in insulin-induced membrane translocation of GLUT4.

### PI(4,5)P_2_ Rescues CFTR Related Defective GLUT4 Translocation

**FIGURE 3:**
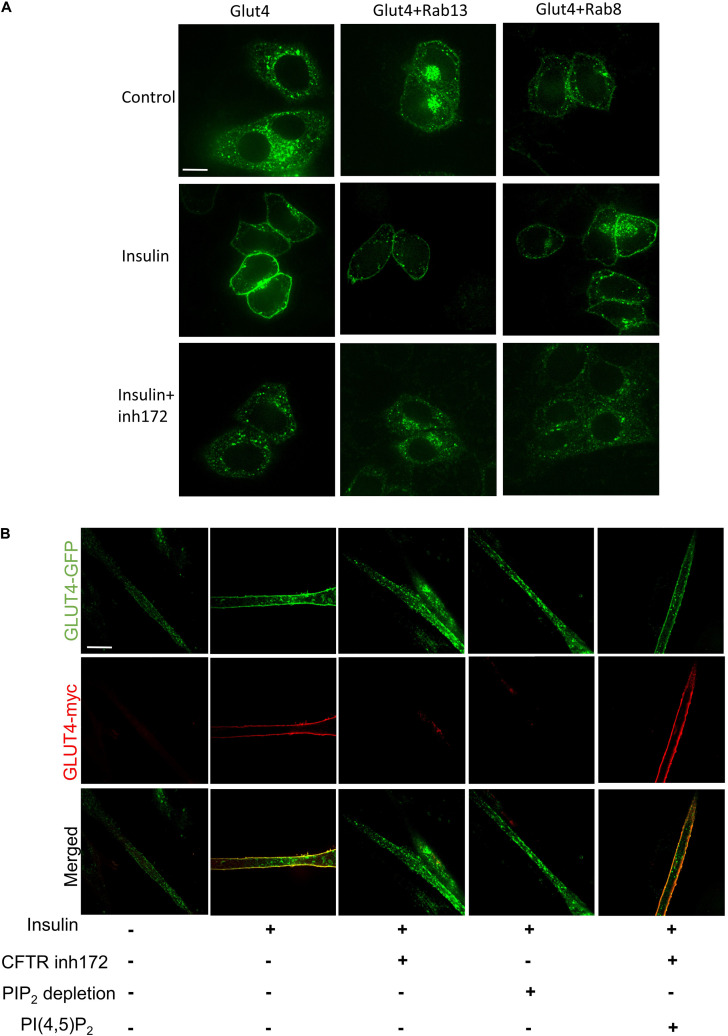
PI(4,5)P_2_ rescues CFTR related defective GLUT4 translocation. **(A)** The membrane localization of GLUT4 in Hela cells after overexpression of Rab8 and Rab13 was detected by confocal microscopy. Glut4 (green), Scale bar, 10 μm. **(B)** C2C12 myotubes were treated with insulin, CFTR inh172, PIP_2_ depletion agents, or PI(4,5)P_2_. PIP_2_ depletion agents are consist of PI_3_K inhibitor (1 μM wortmanin) and PLC agonist (50 μM m-3M3FBS). GLUT4 translocation to the plasma membrane was detected by confocal microscopy. Glut4-GFP (green), Glut4-myc (red). Scale bar, 10 μm.

Several studies indicated that Rab8A and Rab13 could also contribute to the translocation of GLUT4 (Sano et al., 2007; Randhawa et al., 2008; Sun et al., 2010). Rab8A or Rab13 was co-transfected with myc-GLUT4-EGFP in Hela cells; we found that Rab8A or Rab13 could not rescue GLUT4 membrane translocation under CFTR (inh)-172 treatment (Figure 3A), suggesting Rab8A or Rab13 is not involved in this process. PI(4,5)P_2_ mediated insulin-induced actin remodeling is another crucial component in GLUT4 translocation (Kanzaki and Pessin, 2001; Kanzaki et al., 2004; Leto and Saltiel, 2012; Lim et al., 2015). To identify the role of PI(4,5)P_2_ in the process of GLUT4 translocation, C2C12 myotubes were transiently transfected with myc–GLUT4–EGFP fusion plasmids. Insulin-induced GLUT4 translocation in C2C12 myotubes was blocked after endogenous PI(4,5)P_2_ was depleted by application of both PI_3_K inhibitor (wortmanin) and PLC agonist (Figure 3B). The defective GLUT4 membrane translocation induced by CFTR (inh)-172 could also be rescued by exogenous PI(4,5)P_2_ (Figure 3B). As CFTR is a chloride channel, we suspected whether the chloride channel property of CFTR was also involved with GLUT4 translocation. By replacing the chloride with arginine in bath solution, we found that the translocation of GLUT4 was still blocked by CFTR (inh)-172 (Supplementary Figure S2A). In conclusion, these results indicated that CFTR regulated GLUT4 membrane translocation by PI(4,5)P_2_.

### CFTR Affects PI(4,5)P_2_ Concentration via PIP5K1A

**FIGURE 4:**
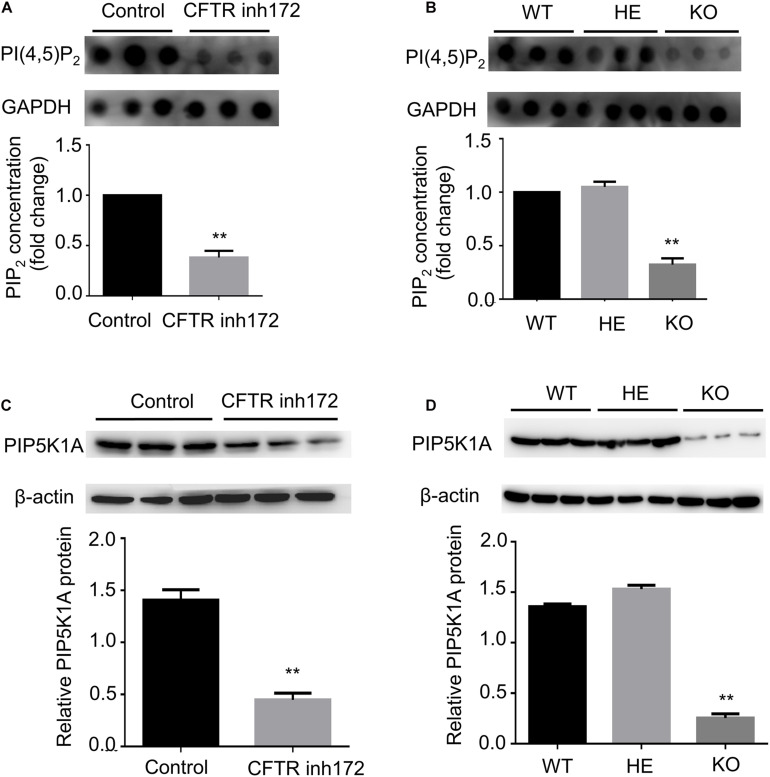
CFTR regulates PIP5KA expression and PI(4,5)P_2_ concentration. **(A)** C2C12 myotubes were treated with CFTR inh172, and the concentration of PI(4,5)P_2_ was measured by dot blot. **P < 0.01 compared with control (*n* = 3). **(B)** The concentration of PI(4,5)P_2_ in mice FDB muscle was measured by dot blot. **P < 0.01 compared with control (*n* = 3). **(C)** C2C12 myotubes were treated with CFTR inh172, and the protein level of PIP5KA was measured by western blot. **P < 0.01 compared with control (*n* = 3). **(D)** The protein level of PIP5KA in mice FDB muscle was measured by western blot. **P < 0.01 compared with control (*n* = 3).

To confirm the speculation that inhibition of CFTR might affect cellular PI(4,5)P_2_ concentration. PI(4,5)P_2_ concentration was detected by dot-blot analysis. C2C12 myotubes were treated with CFTR (inh)-172 for 24 h, and we found that CFTR (inh)-172 could significantly down-regulate PI(4,5)P_2_ concentration (Figure 4A). We also measured the concentration of PI(4,5)P_2_ in FDB muscle fibers isolated from CFTR WT, HET, and KO mice. The concentration of PI(4,5)P_2_ in CFTR^–/–^ mice FDB muscle fibers was significantly lower than WT and HET mice (Figure 4B). PIP5K1A induces the phosphorylation of phosphatidylinositol 4-phosphate (PtdIns4P) to generation PI(4,5)P_2_. Studies indicated that that PI(4,5)P_2_ generation by PIP5K1A was associated with GLUT4 vesicle recycling (Kanzaki et al., 2004) and autophagic lysosome reformation (Rong et al., 2012). To understand how CFTR regulates PI(4,5)P_2_ expression, the PIP5K1A expression level was measured. We found that CFTR (inh)-172 induced a reduction of PIP5K1A expression in C2C12 myotubes (Figure 4C). The expression of PIP5K1A was also significantly decreased in CFTR^–/–^ FDB muscle fibers (Figure 4D). These results suggest that the effect of CFTR on PI(4,5)P_2_ concentration is via the regulation of PIP5K1A expression.

## Discussion

In this study, we discovered a previously unrecognized regulation effect of CFTR to the GLUT4 cellular translocation, which provides a new perspective on the mechanism of CFRD. It was found that CFTR^–/–^ mice exhibited normal insulin secretion but reduced insulin sensitivity and glucose tolerance. Insulin facilitates the entry of glucose into muscle through GLUT4. Meanwhile, Insulin-induced GLUT4 translocation to the cell membrane was also blocked in CFTR^–/–^ mice, suggesting glucose homeostasis alteration from defective intracellular GLUT4 transportation. Unexpectedly, PI(4,5)P_2_ could rescue the effect of CFTR deficiency. Furthermore, it was found that CFTR deficiency leads to a significantly decreased expression of PIP5K1A, which reflected on PI(4,5)P_2_ concentration. Taken together, we discovered a new signal pathway of CFTR/GLUT4, which helps explain the glucose homeostasis disorder in CF patients.

To explain CFRD, several studies have shown that CFTR played a vital role in the regulation of insulin secretion and β cell function (Tofe et al., 2005; Noronha et al., 2011; Konrad et al., 2013; Edlund et al., 2014; Guo et al., 2014). Some studies suggested that insulin resistance was not an essential determinant in CFRD compared with insulin secretion (Cucinotta et al., 1994; Yung et al., 2002). Other studies proved that insulin resistance was highly involved in CFRD, and impaired insulin responses were also reported in CF patients (Hardin et al., 1997; Hardin et al., 2001; Fontes et al., 2015). Our data show that insulin secretion is normal in CFTR^–/–^ mice (Figure 1C); the problem is insulin sensitivity (Figure 1A) and glucose absorption (Figure 1B). Consistent with previous studies (Paroni et al., 2013; Fontes et al., 2015), we also found that the body weight was lower in CFTR^–/–^ mice than WT and HET controls (Figure 1D). Gradual pancreatic destruction in cystic fibrosis causes progressive insulin deficiency. Our results show that in the early stage of CFRD, insulin secretion is expected, so insulin deficiency may not be due to the destruction of pancreatic islets, leading to insufficient insulin secretion, but because CFTR dysfunction affects the insulin-responsive tissues, which manifests as insulin deficiency. In summary, we believe that CFTR affects the pancreas functionality and the insulin-responsive tissues; both contribute to cystic fibrosis-related diabetes (CFRD).

How does CFTR knock out affected insulin sensitivity and glucose tolerance? By binding and activating its cell-surface receptor, insulin triggers signaling cascades that regulate many cellular processes, including stimulating the muscle to dispose of dietary glucose (Richter and Hargreaves, 2013). Insulin stimulation of glucose uptake into muscle depends on the mobilization of GLUT4 to the plasma membrane (Lauritzen et al., 2010; Klip et al., 2014). As Akt2 is an essential kinase in insulin resistance (Cho et al., 2001; McCurdy and Cartee, 2005), we investigate the phosphorylation of Akt2 in CFTR^–/–^ mice. Surprisingly, CFTR deficiency showed no connection with phosphorylation of Akt2 and its downstream protein AS160 (data not shown). Furthermore, other regulators, such as Rab8A and Rab13, also showed no significant impact on CFTR deficiency-induced GLUT4 translocation (Figure 3A). As a result, we focused on the “railway” of protein trafficking in the cell, and studies showed that actin remodeling also played an essential regulatory role in insulin-induced GLUT4 translocation (Kanzaki and Pessin, 2001; Tong et al., 2001). The role of actin dynamic regulated by PI(4,5)P_2_ in GLUT4 translocation has been proved by recent studies (Kanzaki and Pessin, 2001; Kanzaki et al., 2004; Leto and Saltiel, 2012; Lim et al., 2015). Our data show that the depletion of PI(4,5)P_2_ blocks GLUT4 trafficking to the cell membrane, and exogenous PI(4,5)P_2_ can rescue the effect of CFTR deficiency. Thus, we conclude that CFTR in GLUT4 translocation regulation is mediated by PI(4,5)P_2_. In addition to insulin-stimulation, the translocation of GLUT4 to the plasma membrane is also affected by skeletal muscle contraction. Whether CFTR has a role in skeletal muscle contraction needs further clarification.

As CFTR is an ion channel, which makes no sense in catalyzing PI(4,5)P_2_ generation. We proved that the chloride channel property of CFTR was not involved with GLUT4 translocation; only the protein itself is involved in translocation. Therefore, we speculated that CFTR allosteric affects some kinase proteins in the regulation of PI(4,5)P2 generation. The differential gene expression induced by CFTR deficiency has been reported by several studies (Ichikawa et al., 2000; Ogilvie et al., 2011; Chen et al., 2012). We suspected that inhibition or knockdown of CFTR might lead to decreased PIP5K1A expression, which regulates PI(4,5)P_2_ generation. We found that the expression of PIP5K1A in CFTR^–/–^ mice and CFTR (inh)-172 treated C2C12 was significantly reduced, causing lower PI(4,5)P_2_ concentration. In conclusion, PIP5K1A is one of the regulators between CFTR and PI(4,5)P_2_, but further investigation is needed to assess how CFTR deficiency regulates PIP5K1A expression.

In summary, our results suggest a new mechanism of glucose homeostasis disorder in CF patients. These findings identify the role of GLUT4 translocation in CFTR related glucose homeostasis disorder in CF patients, which provides a new potential treatment strategy.

## Data Availability Statement

The original contributions presented in the study are included in the article/Supplementary Material, further inquiries can be directed to the corresponding author/s.

## Ethics Statement

The animal study was reviewed and approved by the University Ethics Committee, Institute of Molecular Medicine, Peking University.

## Author Contributions

YG and LW conceived and coordinated the study. JG and LW conducted molecular experiments and wrote the manuscript. WZ and JG performed the imaging-related experiments. All authors made critical contributions to data analysis, interpretation, discussion, and manuscript preparation and approved the final version.

## Conflict of Interest

The authors declare that the research was conducted in the absence of any commercial or financial relationships that could be construed as a potential conflict of interest.

## Supplementary Material

Supplementary Figure 1 Insulin-induced GLUT4 translocation in HEPG2 and Hela cells.

Supplementary Figure 2 Chloride channel property of CFTR and insulin-induced GLUT4 translocation.

## Abbreviations

CF: Cystic Fibrosis
CFTR: Cystic Fibrosis Transmembrane Conductance Regulator
CFRD: Cystic Fibrosis related diabetes
GLUT4: Glucose Transporter 4.
