# Supplementary material for: CFTR Deficiency Affects Glucose Homeostasis via Regulating GLUT4 Plasma Membrane Transportation
Source: Front Cell Dev Biol. 2021 Feb 15;9:630654. doi: 10.3389/fcell.2021.630654 (PMC7917208; doi:10.3389/fcell.2021.630654)
Supplement: Supplementary Figure 1 — Insulin-induced GLUT4 translocation in HEPG2 and Hela cells. [file Table_1.DOCX]

Supplementary Material

## Supplementary Figures


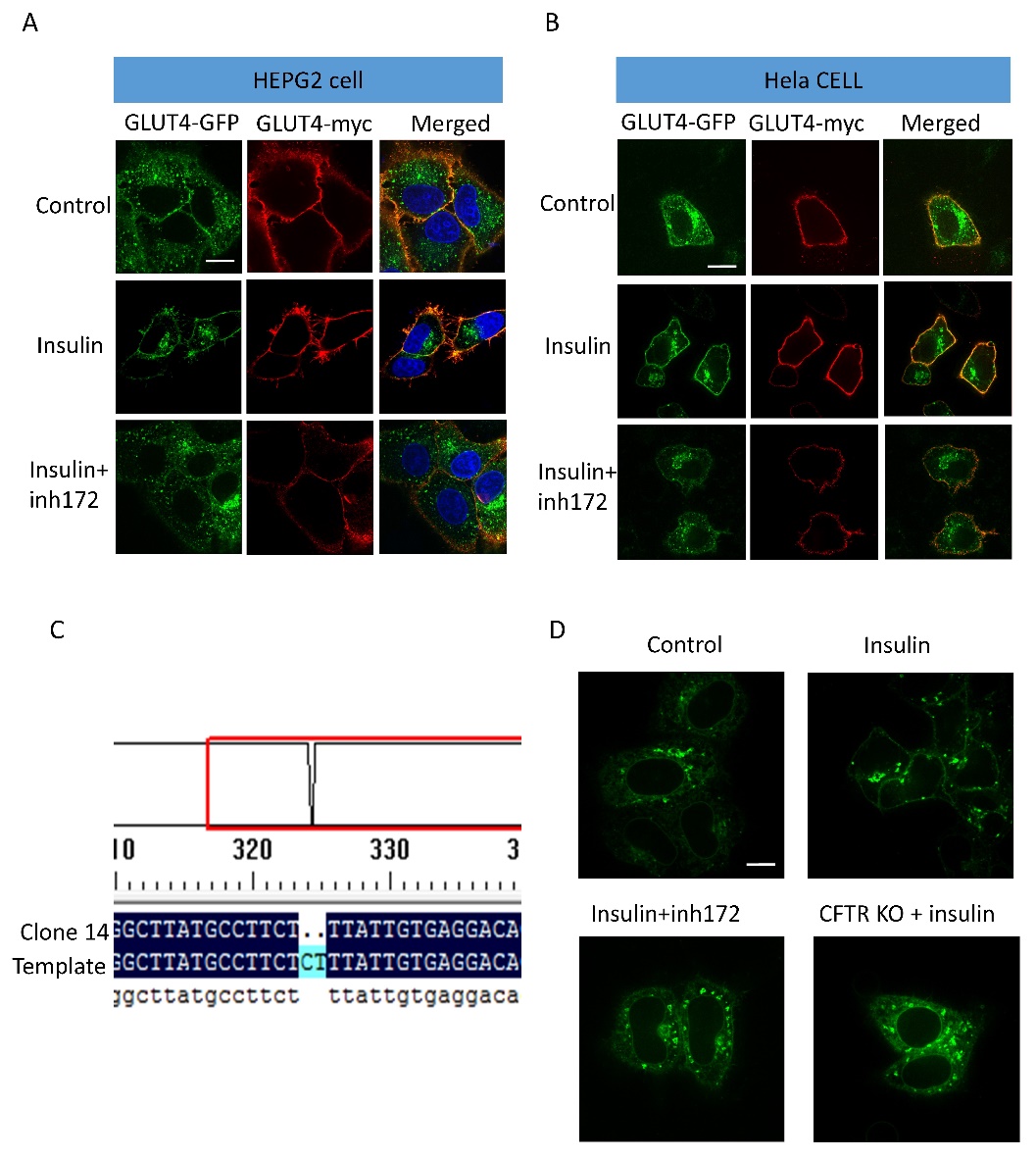


**Supplementary Figure 1.** The membrane localization of GLUT4 in HEPG2 cells and Hela cells. (A)HEPG2 cells after 100 nM insulin treatment were detected by confocal microscopy. Glut4-GFP(green), Glut4-myc(red). Scale bar, 10μm. (B)Hela cells after 100 nM insulin treatment were detected by confocal microscopy. Glut4-GFP(green), Glut4-myc(red). Scale bar, 10μm. (C)CFTR was knock out in Hela cells by CRISPR/Case9, and the knockout of CFTR was detected by sequencing. (D)GLUT4 translocation to the plasma membrane of live Hela cell and Hela-CFTR-/- cells was detected by confocal microscopy. Glut4- GFP(green). Scale bar, 10μm.


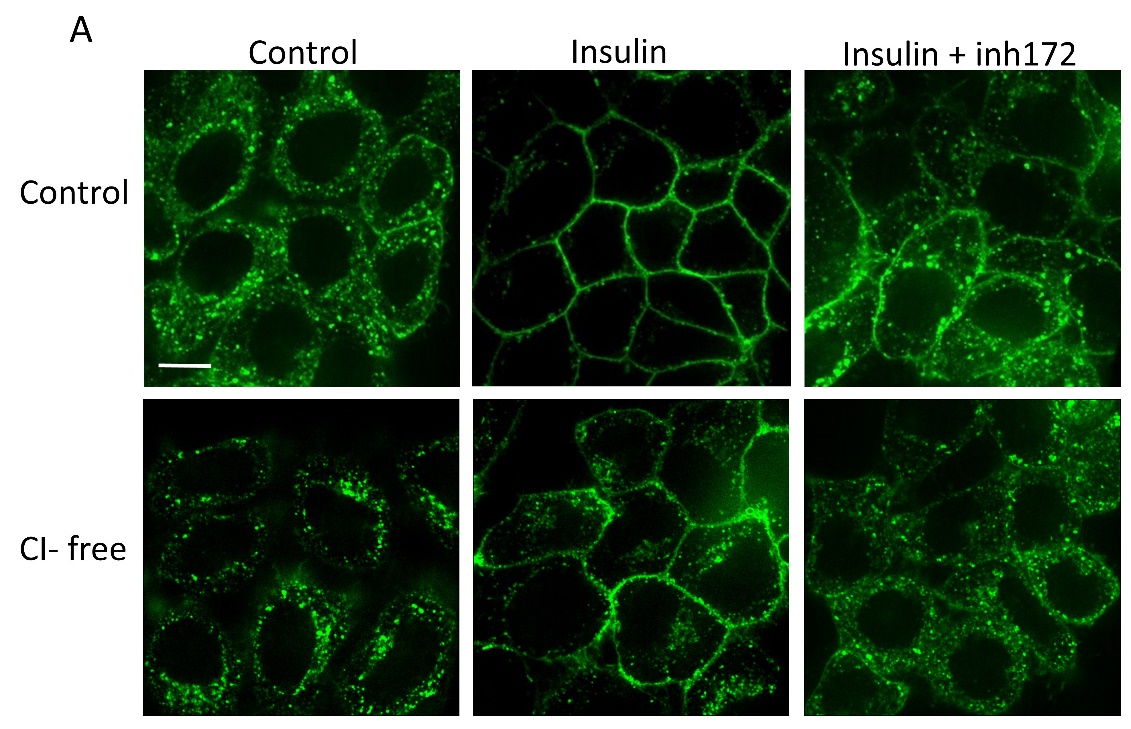


**Supplementary Figure 2.** Regulators of insulin-induced GLUT4 translocation. (A) Hela cells were cultured with CI- free bath solution, and then the membrane localization of GLUT4 was detected by confocal microscopy. Glut4- GFP (green), Scale bar, 10μm.
