# Supplementary material for: CFTR Deficiency Affects Glucose Homeostasis via Regulating GLUT4 Plasma Membrane Transportation
Source: Front Cell Dev Biol. 2021 Feb 15;9:630654. doi: 10.3389/fcell.2021.630654 (PMC7917208; doi:10.3389/fcell.2021.630654)

Fig2A uncropped gel

Glut 4

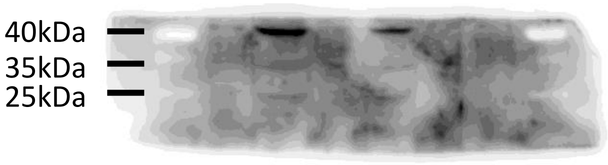

ATP1A1

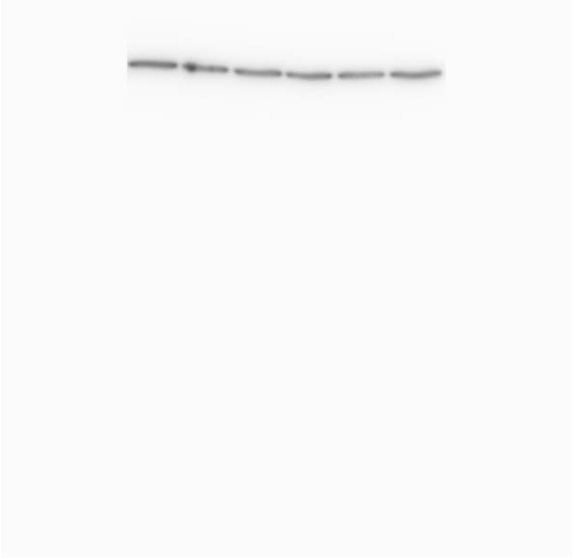

Fig2B uncropped gel

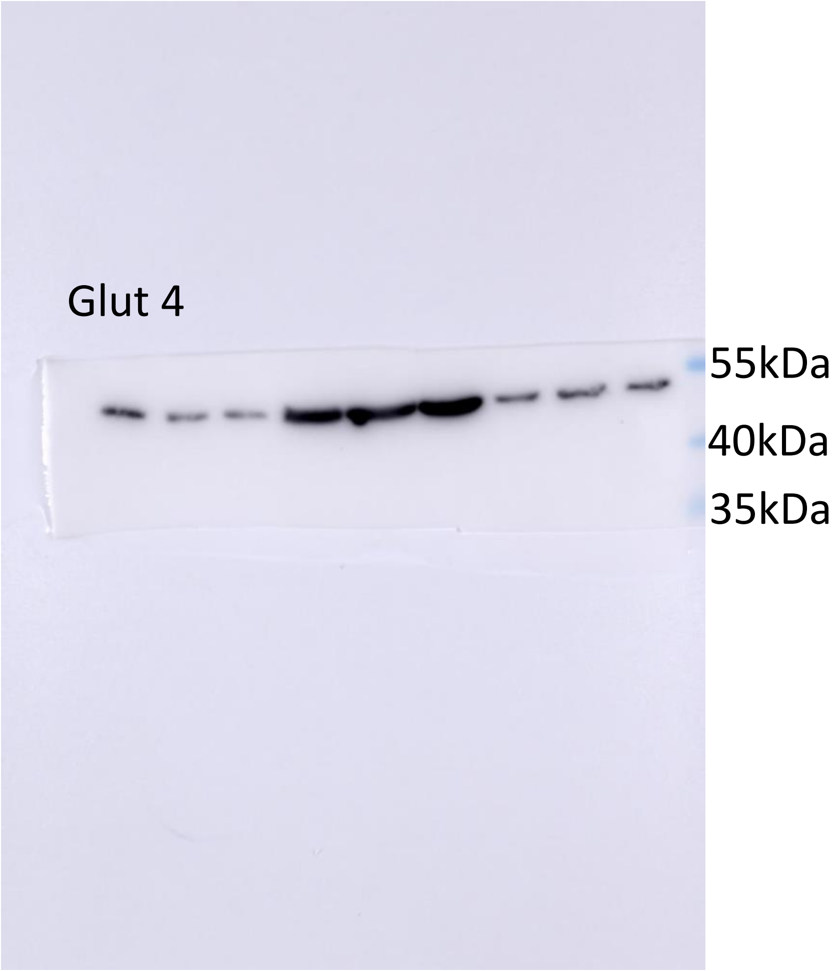

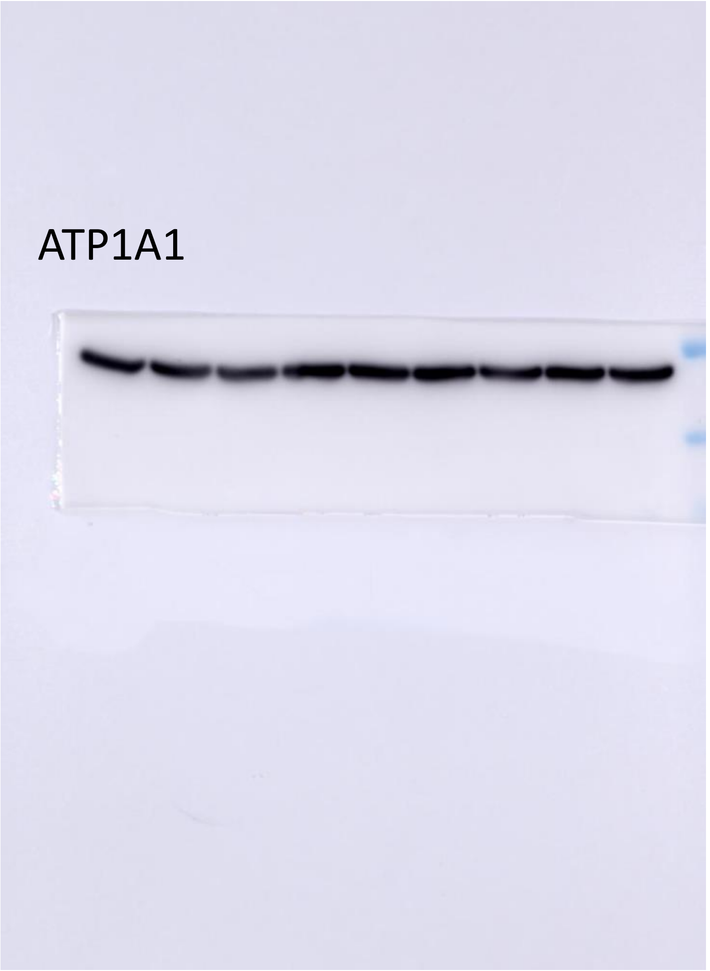

Fig4A uncropped gel

$PI(4,5)P_2$

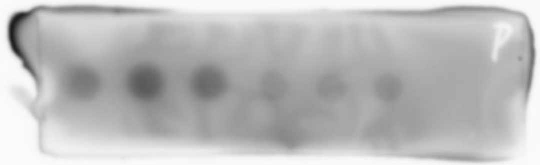

GAPDH

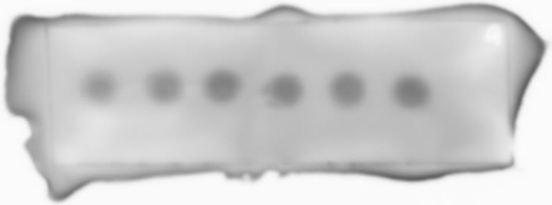

Fig4B uncropped gel

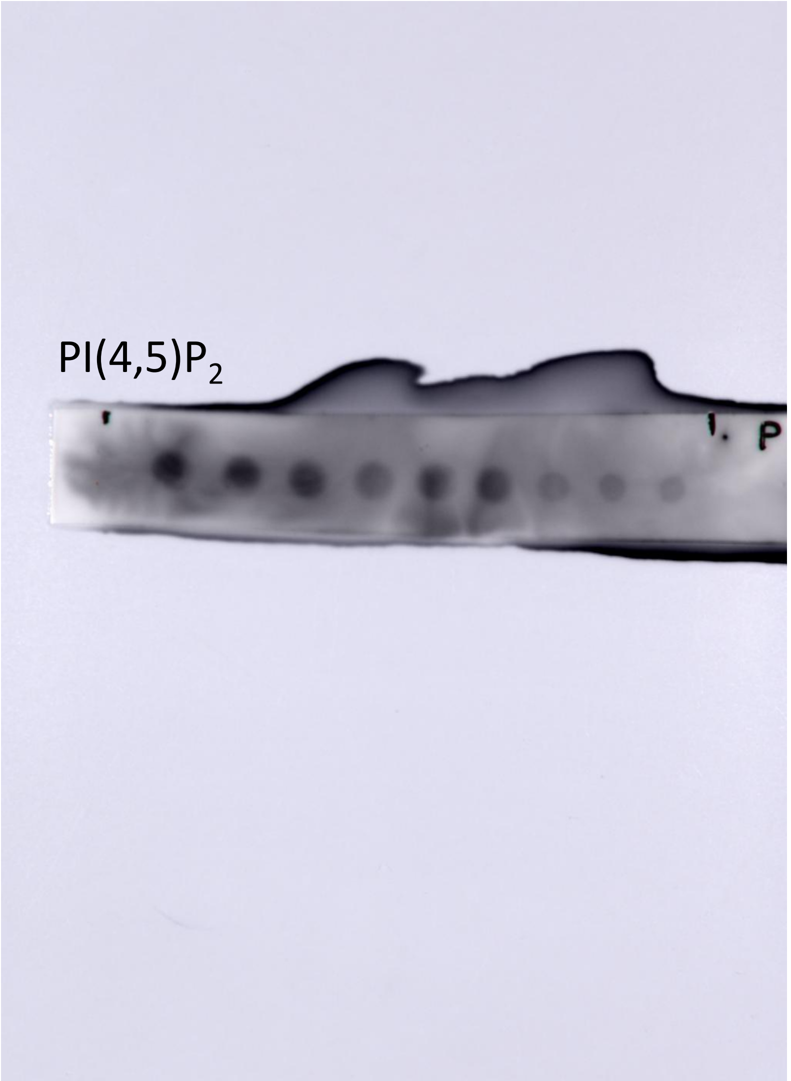

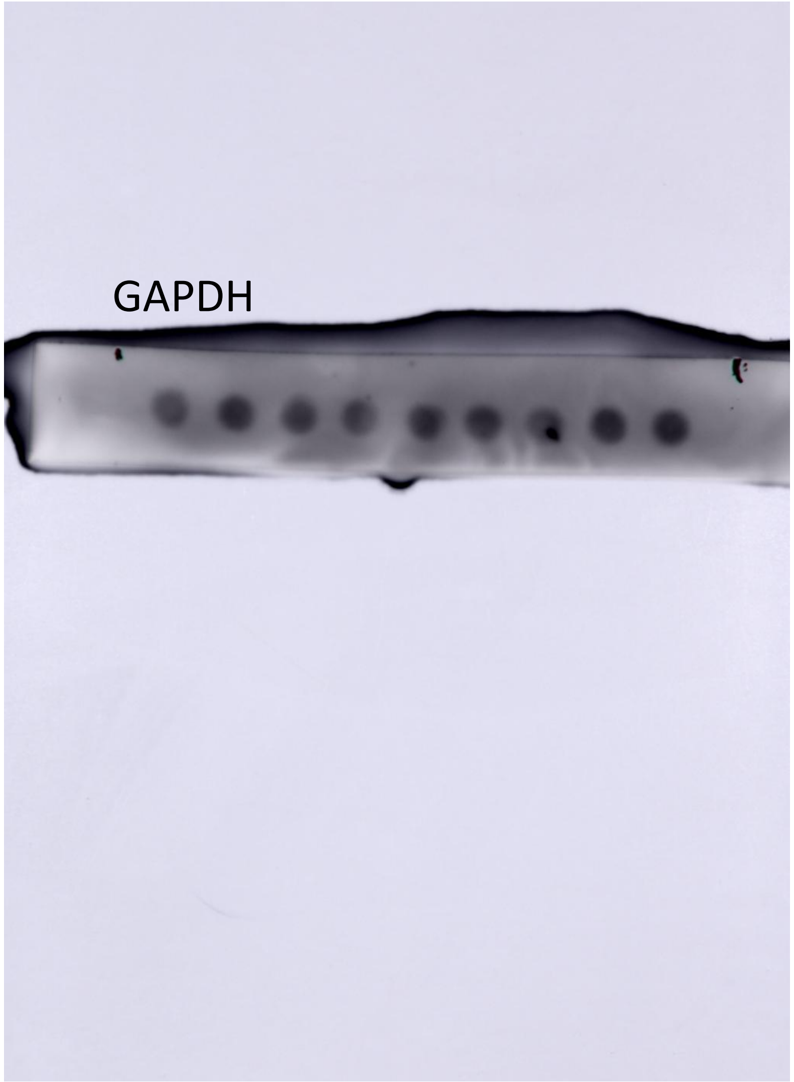

Fig4C uncropped gel

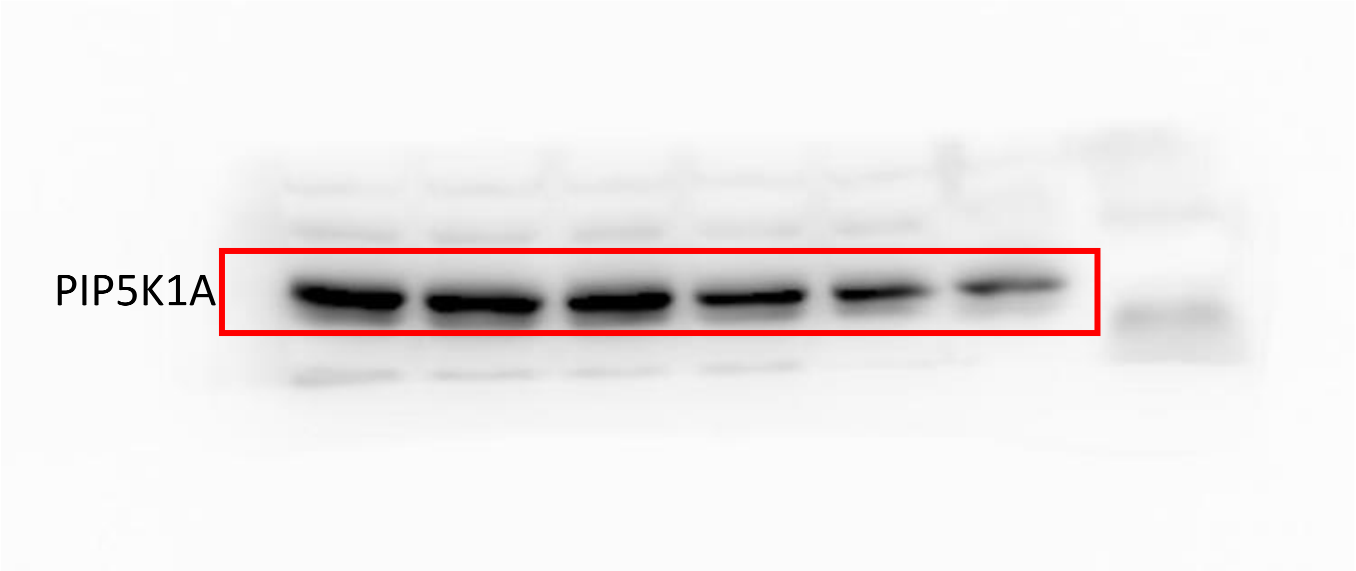

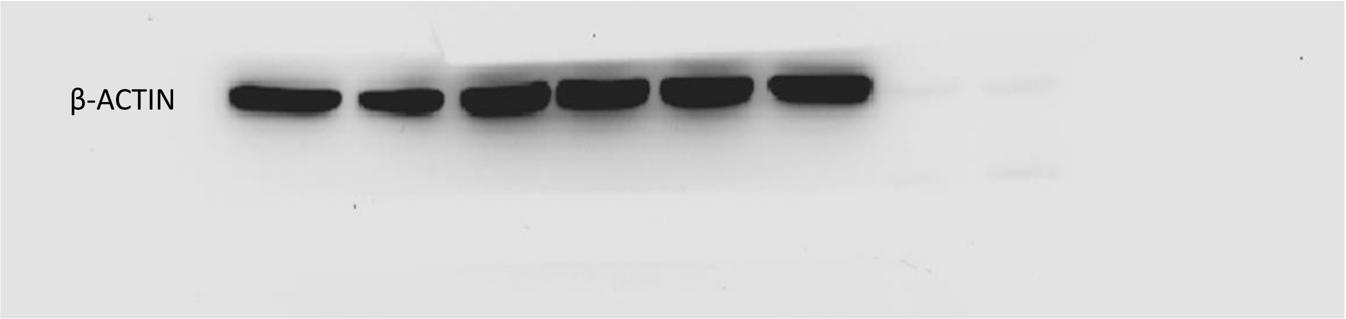

Fig4D uncropped gel

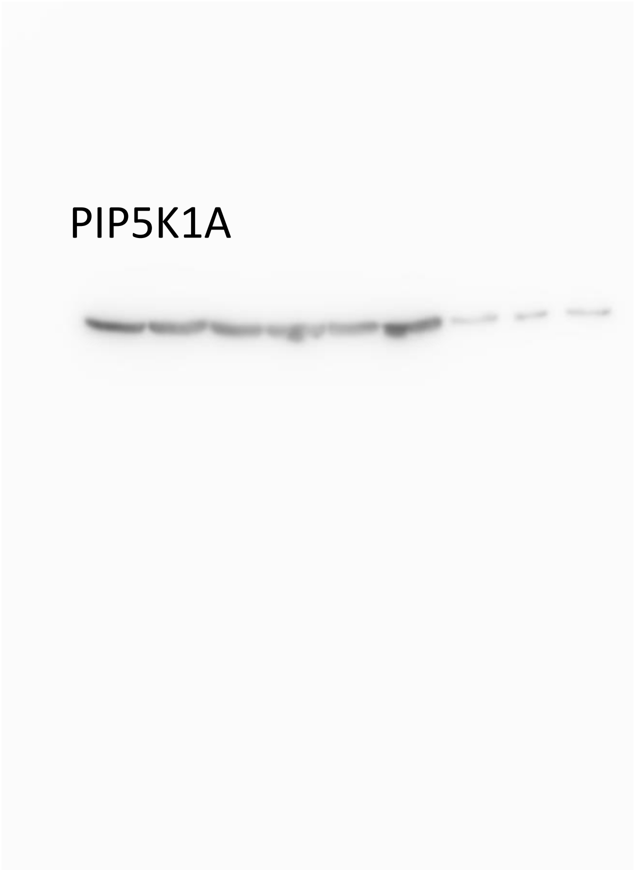

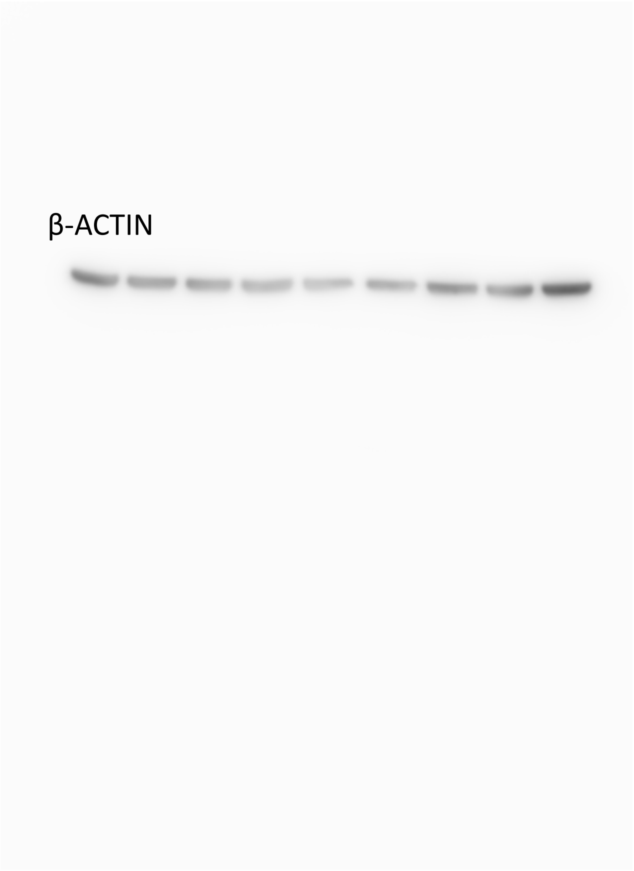

Supplement: Supplementary file 2 [file Data_Sheet_1.PDF]
